# Improved influenza A whole-genome sequencing protocol

**DOI:** 10.3389/fcimb.2024.1497278

**Published:** 2024-11-28

**Authors:** Iryna V. Goraichuk, Jacquline Risalvato, Mary Pantin-Jackwood, David L. Suarez

**Affiliations:** Southeast Poultry Research Laboratory, U.S. National Poultry Research Center, Agriculture Research Service, U.S Department of Agriculture, Athens, GA, United States

**Keywords:** next-generation sequencing, NGS, nanopore, MinION, Illumina, influenza, WGS, RT-PCR

## Abstract

Influenza A virus poses significant public health challenges due to its high mutation rate and zoonotic potential. Whole-genome sequencing (WGS) is crucial for monitoring and characterizing these viruses. Oxford Nanopore Technologies (ONT) and Illumina next-generation sequencing platforms are commonly used, with ONT being advantageous for its long-read capabilities, portability, and unique ability to access raw data in real-time during sequencing, making it suitable for rapid outbreak responses. This study optimizes the ONT Ligation Sequencing Influenza A Whole Genome protocol by refining RT-PCR kits, primers, and purification methods, and evaluating automation for high-throughput processing. The alternative RT-PCR kits, combined with alternative primers, significantly improved read depth coverage and reduced short, untargeted reads compared to the original ONT protocol. The improvement was particularly evident in the minimum read depth coverage of polymerase segments, which often face challenges with achieving uniform coverage, displaying higher coverage at the 5’ and 3’ termini, and lower coverage in the central regions. This optimized protocol for targeted influenza A WGS not only enhances sequencing quality and efficiency, but is applicable to all NGS platforms, making it highly valuable for studying influenza adaptation and improving surveillance. Additionally, this protocol can be further refined and adapted for the sequencing of other pathogens, broadening its utility in various pathogen monitoring and response efforts.

## 1 Introduction

Influenza A virus is a major pathogen responsible for seasonal flu epidemics and occasional human pandemics, posing significant public health challenges globally (Spackman, 2014; Suarez, 2017; Swayne et al., 2020). The high mutation rate of Influenza A, coupled with its ability to infect various host species, including birds and mammals, makes it a constant threat to public health. The rapid evolution and genetic diversity of influenza viruses necessitate continuous surveillance and in-depth genetic characterization to monitor emerging strains and understand their zoonotic potential.

Whole genome sequencing (WGS) has emerged as a crucial tool for the comprehensive analysis of influenza virus genomes (Croville et al., 2018; Keller et al., 2018; King et al., 2020; Van Poelvoorde et al., 2020; Crossley et al., 2021; Chauhan and Gordon, 2022; Min et al., 2022; Andrés et al., 2023; Nabeshima et al., 2023; Croville et al., 2024). Traditional Sanger sequencing methods have largely been replaced by next-generation sequencing (NGS) technologies, which allow for high-throughput and cost-effective sequencing of complete viral genomes (McGinn and Gut, 2013). Among NGS platforms, Oxford Nanopore Technologies (ONT) and Illumina are commonly used for influenza virus sequencing due to their ability to generate long and short reads, respectively (Lee, 2020). Illumina sequencing, known for its high accuracy and short reads, has been extensively used for influenza virus WGS (Rutvisuttinunt et al., 2013; Mitchell et al., 2021; Galli et al., 2022; Wang et al., 2024). However, the ONT platform is particularly advantageous for sequencing full-length viral genomes due to its capability of producing long sequence reads, simplifying the assembly process and providing more accurate genome reconstruction (Deamer et al., 2016; MacKenzie and Argyropoulos, 2023).

Both NGS platforms can be used for targeted amplicon and untargeted random sequencing (Rutvisuttinunt et al., 2013; Goraichuk et al., 2017; Lewandowski et al., 2019; Poen et al., 2020; Goraichuk et al., 2023; Goraichuk et al., 2024a; Kuchinski et al., 2024). Random sequencing (or untargeted sequencing) captures a comprehensive snapshot of all nucleic acids present in a sample without prior knowledge of the target sequences, enabling the detection of novel or unexpected pathogens and providing a more complete picture of the viral genome, including non-coding regions and structural variations. Furthermore, it can reveal co-infections and the presence of other microorganisms in the sample, offering insights into the microbial community and potential interactions (Kariithi et al., 2023; Lu et al., 2024). Random sequencing is also less biased by primer design, allowing for more uniform coverage across the entire genome (Aird et al., 2011). However, random amplification in diagnostic samples will also amplify host rRNA, often a high percentage of the total reads, which can greatly decrease the sensitivity of detection of important pathogens (Parris et al., 2022).

Targeted amplicon sequencing, in comparison to untargeted random sequencing, provides several significant advantages and is particularly suited for specific applications. Utilizing specific primers ensures that only the targeted regions are amplified and sequenced, significantly reducing contamination from the host and other abundant untargeted bacterial DNA/RNA. This targeted approach drastically amplifies regions of interest, allowing for much higher sequencing depth and higher sensitivity, which is crucial for detecting low-frequency mutations and single nucleotide polymorphisms (SNPs) (Dinis et al., 2016; Takayama et al., 2021).

For influenza viruses, a targeted amplicon approach can amplify the whole genome by leveraging the conserved termini of influenza segments (Hoffmann et al., 2001). This method uses the highly conserved sequences at the 5’ and 3’ ends of each segment to design primers that can universally amplify all eight segments in a single RT-PCR reaction. This technique simplifies the workflow and reduces the time and cost associated with sample preparation, which is especially important in outbreak situations (Zhou et al., 2009).

After amplification, the required read depth coverage can be achieved in a shorter time due to the high number of amplicons produced. This efficiency makes the ONT platform particularly advantageous. The unique capability of ONT sequencers to access raw sequencing data in real-time during the run and to terminate sequencing runs once sufficient data has been collected, allows for rapid completion of sequencing, often within a few hours. This contrasts with the extended runs required by some high-throughput Illumina instruments, such as the MiSeq, which can take several days to complete. The ability of near real-time sequencing of ONT, coupled with its portable size, not only accelerates the overall workflow but also allows for more efficient use of sequencing resources. This is particularly critical in time-sensitive scenarios such as outbreak investigations or clinical diagnostics, where rapid turnaround times are essential for effective response and management.

However, challenges remain in achieving consistent and comprehensive read depth coverage across all genomic segments. Studies have demonstrated the effectiveness of ONT sequencing in capturing the full-length influenza A genome, albeit with challenges in achieving uniform read depth coverage across all segments, particularly the longer polymerase segments (PB2, PB1, and PA) (Van den Hoecke et al., 2015; Wang et al., 2015; Lee et al., 2016; Miah et al., 2023). These segments often exhibit U-shaped read coverage patterns, characterized by higher coverage at the 5’ and 3’ termini and lower coverage in the central regions. This pattern is partly due to the presence of defective interfering particles (DIPs), a common feature of influenza viruses, which are shorter sequences derived mainly from the polymerase segments (Davis and Nayak, 1979; Davis et al., 1980; Saira et al., 2013; Alnaji et al., 2019; Ferreri et al., 2019; Świętoń et al., 2020; Ziegler and Botten, 2020).

While shorter reads can be bioinformatically filtered post-sequencing to address the U-shaped coverage issue, this approach does not mitigate the initial sequencing limitation. The presence of numerous short reads during sequencing compromises the ability to capture longer, complete-length segments, leading to insufficient read depth coverage for these critical regions. Therefore, it is crucial to eliminate short reads before sequencing to ensure a higher minimum depth coverage for longer reads, thereby enhancing the reliability of downstream analysis.

To address these challenges, we optimized the ONT Ligation Sequencing Influenza A Whole Genome V14 protocol (Oxford_Nanopore_Technologies, 2024) by refining the use of RT-PCR kits, primer sets, and purification methods, and additionally evaluating the feasibility of automation for high-throughput sample processing. The refined method can also be used for amplicon-based sequencing on other NGS platforms.

## 2 Materials and methods

### 2.1 Samples

Eight avian influenza isolates of varying virulence and subtypes (**Table 1**) from the Southeast Poultry Research Laboratory (SEPRL) were used to validate the performance of the updated protocol for influenza A whole genome sequencing. Viruses were propagated in 9–11-day-old specific-pathogen-free (SPF) embryonated chicken eggs. The harvested allantoic fluids were subsequently used in this study. Background information on the egg-grown isolates is summarized in **Table 1**.

**Table 1:** Background information on influenza A viruses used in this study.

Isolate ID	Host	Country	Year of collection	Pathogenicity	Subtype	GenBank
F12505B	Chicken	Egypt	2016	HPAIV ^1^	H5N1	PQ064247 - PQ064254
MX/37905	Chicken	Mexico	2015	HPAIV	H7N3	PQ106540 - PQ106540, MH342039
NSW/3121-1	Chicken	Australia	2012	HPAIV	H7N7	PQ064551 - PQ064558
1158-11406-1	Chicken	England	2008	HPAIV	H7N7	PQ064115 - PQ064122
PA/35154	Chicken	USA	1991	LPAIV ^2^	H1N1	EU735794 - EU735801
TX/G021090002	Chicken	USA	2002	LPAIV	H5N3	PQ064267 - PQ064274
CA/K0301417	Chicken	USA	2003	LPAIV	H6N2	PQ064136 - PQ064143
CO/169118-13	Turkey	USA	2002	LPAIV	H8N4	GU051913 - GU051917, PQ060363 - PQ060365

^1^Highly pathogenic avian influenza virus; ^2^Low pathogenic avian influenza virus.

### 2.2 RNA extraction and RT-qPCR

Total RNA was extracted from infectious allantoic fluids using the MagMAX™-96 AI/ND Viral RNA Isolation Kit (Applied Biosystems, USA) following the manufacturer’s instructions. RNA quality and concentrations were assessed using the EzDrop 1000C spectrophotometer (Blue-Ray Biotech, Taiwan). The presence of influenza RNA was confirmed using the NVSL avian influenza matrix gene RT-qPCR assay, as previously described (Spackman et al., 2002; Goraichuk et al., 2024a). Extracted viral RNA were then used for the comparison of different RT-PCR kits, primer sets, and purification methods.

### 2.3 RT-PCR kit comparison

To increase the minimum read depth coverage of polymerase segments, we compared the performance of three different RT-PCR kits for the simultaneous amplification of influenza A genome segments. Multisegment RT-PCR amplification was performed according to the ONT Ligation Sequencing Influenza A Whole Genome V14 protocol (Oxford_Nanopore_Technologies, 2024) using the recommended RT-PCR kit (SuperScript™ III One-Step RT-PCR System with Platinum™ *Taq* DNA Polymerase, Invitrogen, USA) and compared to two other RT-PCR kits with alternative RT and DNA polymerases (SuperScript™ IV One-Step RT-PCR System, Invitrogen, USA and LunaScript^®^ Multiplex One-Step RT-PCR Kit, New England Biolabs, USA), referred to as SSIII, SSIV, and LS, respectively. The key specifications of the three RT-PCR kits are summarized in **Table 2**. Thermocycling conditions and reaction volumes for the ONT-recommended SSIII RT-PCR were performed as described in the ONT Ligation Sequencing Influenza A Whole Genome V14 protocol (Oxford_Nanopore_Technologies, 2024). For the alternative SSIV and LS RT-PCR kits, conditions were adjusted according to the manufacturers’ recommendations and the detailed optimized protocols have been deposited at protocol.io: dx.doi.org/10.17504/protocols.io.bp2l62r15gqe/v1 (Goraichuk et al., 2024b).

**Table 2:** Comparison of RT-PCR kits specifications.

Parameter	ONT-recommended SSIII	Alternative SSIV	Alternative LS
RT-PCR Kit	SuperScript III One-Step RT-PCR System with Platinum *Taq* DNA Polymerase (Invitrogen)	SuperScript IV One-Step RT-PCR System (Invitrogen)	LunaScript Multiplex One-Step RT-PCR Kit (New England Biolabs)
One-step RT-PCR	Yes	Yes	Yes
Reverse Transcriptase	SuperScript III	SuperScript IV	Luna Warm Start
Recommended RT Time	15-30 min	10 min	10 min
DNA Polymerase	Platinum *Taq*	Platinum SuperFi	Q5 Hot Start High-Fidelity
Fidelity (vs. *Taq*)	1X	300X	280X
Hot-start Temperature	94°C	98°C	98°C
GC-Rich PCR Performance	High	High	High
Optimal amplicon length	200 - 4,500 bp	Up to 13.8 kb	100 - 1,500 bp
Price per sample	$8.62	$9.71	$3.86

### 2.4 Primers comparison

Following the RT-PCR kit comparison, the performance of two primer sets was evaluated for the simultaneous amplification of influenza A genome segments to determine which provided higher minimum read depth coverage in polymerase segments. All eight gene segments were amplified using the ONT-recommended Tuni primer set (Zhou et al., 2009) and the alternative Opti primer set (Mena et al., 2016; Leyson et al., 2019) with both the ONT-recommended SSIII and the alternative SSIV RT-PCR kits. Both Tuni and Opti primer sets incorporate influenza Uni 12 and Uni 13 conserved termini at the end of all 8 genomic segments, along with a 10 nt tail at the 5’ end to enhance PCR amplification. The primary difference between the primer sets lies in nucleotide compositions of the 5’ tails, which necessitated adjustments in the annealing temperatures. The sequences of the primers, master mix compositions, and thermocycling conditions are summarized in **Supplementary Table 1**. After thermocycling, 5 μL of the product was visualized on a 1.5% agarose gel to verify the amplification of all genomic segments. Additionally, the concentration and purity of the amplicons were measured using the EzDrop 1000C spectrophotometer (Blue-Ray Biotech, Taiwan), Qubit 1X dsDNA High Sensitivity Kit on a Qubit 4 fluorometer (Invitrogen, USA), and High Sensitivity D5000 ScreenTape on a 4150 TapeStation (Agilent Technologies, USA).

### 2.5 Purification comparison

To evaluate the effectiveness of different amplicon purification kits in reducing short untargeted reads, we compared two magnetic bead-based and two column-based purification kits. After amplification using the SSIV RT-PCR kit and the Opti primer set, amplicons were purified according to the Nanopore protocol using the bead-based Kit 1 (Agencourt AMPure XP beads, Beckman Coulter, USA) at a 1:1 bead:sample ratio. This was then compared to three alternative purification kits: another magnetic bead-based Kit 2 (ChargeSwitch PCR Clean-Up Kit, Invitrogen, USA) and two column-based kits (Kit 3 – PureLink PCR Purification Kit (Invitrogen, Lithuania) and Kit 4 – Select-a-Size DNA Clean & Concentrator (Zymo Research, USA)) following the manufacturer’s protocols. The elution volumes differed between kits as follows: Kit 1 – 15 µl, Kit 2 - 25 µl, Kit 3 – 50 µl, and Kit 4 – 15 µl.

### 2.6 Purification automation assessment

To improve time efficiency when processing numerous samples, we compared the performance of two bead-based purification methods using both manual and automated processes with the KingFisher Purification System 5400000 (Thermo Scientific, USA). For the automated process, the elution volume was increased to 30 µl according to the manufacturers’ recommendations. Following purification, the concentration and purity of the amplicons were measured using the EzDrop 1000C spectrophotometer (Blue-Ray Biotech, Taiwan), Qubit 1X dsDNA High Sensitivity Kit on a Qubit 4 fluorometer (Invitrogen, USA), and High Sensitivity D5000 ScreenTape on a 4150 TapeStation (Agilent Technologies, USA).

### 2.7 Illumina library preparation and sequencing

To obtain the reference genomes of the viruses used in this study, we conducted two Illumina sequencing runs using sequence-independent, single-primer amplification (SISPA) library preparation methods (Chrzastek et al., 2017), with and without a previously published pretreatment to remove host and bacterial rRNAs (Parris et al., 2022; Bakre et al., 2023; Goraichuk et al., 2024a). Illumina libraries were prepared using the Illumina DNA Prep (Illumina, USA) according to the manufacturer’s recommendations. After quantification using the Qubit 1X dsDNA High Sensitivity Assay Kit (Invitrogen, USA) and High Sensitivity D5000 Screen Tape (Agilent Technologies, USA), the libraries were pooled (4 nM, 10 µl each), spiked with a control library (5% PhiX library v3), diluted to 12 pM final concentration and sequenced (paired-end; 2x300 bp) using the 600-cycle MiSeq Reagent Kit v3 (Illumina, USA) on an Illumina MiSeq instrument.

### 2.8 Nanopore library preparation and sequencing

For the comparison of different RT-PCR kits, primer sets, purification kits, and purification automation methods, four Nanopore sequencing libraries were prepared using the Native Barcoding Kit 24 V14 (SQK-NBD114.24, Oxford Nanopore Technologies, England). A total of 24 samples per comparison were pooled together after barcoding, and the final library was quantified using the High Sensitivity D5000 Screen Tape on a 4150 TapeStation (Agilent Technologies, USA). We then sequenced 20 fmol of the prepared library for initial RT-PCR comparison, as recommended by the ONT protocol. Subsequently, we increased the loading amount up to 80 fmol in the following runs to achieve higher flow cell pore occupancy and longevity of flow cell (**Supplementary Table 2**). All comparisons were performed on separate R10.4.1 MinION flow cells (FLO-MIN114, Oxford Nanopore Technologies, England) using the Mk1C sequencer with the MinKNOW 23.04.8 software. Sequencing was run for ~ 24 hours.

### 2.9 NGS data analysis

The Illumina raw sequencing data was processed within the Galaxy platform. The raw reads from samples prepared using SISPA, both with and without rRNA depletion pretreatment, were merged to enhance the yield of viral reads for complete genome coverage. The forward and reverse raw sequence reads were joined and their quality was assessed using FastQC v0.63 (Andrews, 2023). Low-quality bases were trimmed and short reads were filtered by Fastp 0.32.2 (Chen et al., 2018). Host reads (*Gallus gallus* and *Meleagris gallopavo*) were eliminated using the Burrows-Wheeler Alignment Tool (BWA-MEM) (Li and Durbin, 2009), and the output was sorted using Samtools merge 1.15.1 tool (Danecek et al., 2021). Digital normalization via median k-mer abundance was carried out using the BBTools: BBNorm (Bushnell et al., 2017). The remaining unmapped reads were subjected to *de novo* assembly using the MIRA Assembler v3 (Cock et al., 2013) to obtain an intermediate genome scaffold. The consensus sequence was then re-called by mapping trimmed and filtered paired collection to the genome scaffold using the BWA-MEM (Li and Durbin, 2009). PCR duplicates were removed using RmDup 2.0.1 (Li et al., 2009), and then final consensus sequences were generated using the bam2consensus tool (Volkening, 2023).

The Nanopore raw Pod5 files were basecalled with a high-accuracy algorithm to generate FastQ files, which were then demultiplexed and trimmed using Dorado 7.1.4 within the MinKNOW 23.07.12 (bionic) software on a MinION Mk1C instrument. Reads with a minimum quality of 9 were considered for further analysis. For the RT-PCR kits and primer sets comparisons, short reads below 200 bp were removed during sequencing run. Filtered MinKNOW-generated FastQ files containing 4,000 “pass” reads per file were concatenated into a single consolidated file for each barcoded sample. Further analysis of Nanopore reads was performed on the Galaxy platform. The influenza genome was assembled by aligning filtered reads with reference genomes obtained from Illumina sequencing using minimap2 (Li, 2018) and verified in Geneious Prime 2023.0.1. The coverage of the influenza virus genome was obtained using SAMtools depth (Danecek et al., 2021).

### 2.10 Statistical analysis

GraphPad Prism 10.2.3 (Sović et al., 2016) was used for data visualization and statistical analysis. A one-way ANOVA followed by Tukey’s multiple comparisons test was utilized to compare the relative difference in the total number of sequenced reads, the mean number of reads, and minimum read depth coverage among different RT-PCR kits, primer sets, and purification kits for the eight viruses sequenced. The *p*-value ≤ 0.05 was considered statistically significant.

## 3 Results

### 3.1 Reference Illumina sequencing

Eight selected influenza A samples were confirmed positive by the NVSL avian influenza matrix gene RT-qPCR assay, with Ct values ranging from 11.6 to 17.6 (**Table 3**). Two Illumina MiSeq runs generated 1,022,630 to 1,807,144 total raw paired-end reads per sample. *De novo* assembly resulted in over 99% genome breadth coverage (complete coding genome coverage) of the avian influenza virus in all sequenced samples. The obtained genome sequences were deposited in GenBank under accession numbers PQ060363-PQ060365, PQ064115-PQ064122, PQ064136-PQ064143, PQ064247-PQ064254, PQ064267-PQ064274, PQ064551-PQ064558, PQ106540-PQ106540, EU735794, EU735796, EU735799-EU735801, MH342039 (**Table 1**) and were used as reference genomes in the optimization of Nanopore sequencing.

**Table 3:** Summary of Illumina sequencing.

Isolate	Subtype	RT-qPCR, Ct ^1^	Total Reads	Influenza Reads	Influenza Genome Breadth Coverage, %
A/Ck/Egypt/F12505B/2016	H5N1	17.6	1,798,924	1,161,501	99.92
A/Ck/Mexico/MX/37905/2015	H7N3	11.6	1,706,564	427,046	99.24
A/Ck/Australia/NSW/3121-1/2012	H7N7	15.2	1,060,730	888,279	99.15
A/Ck/England/1158-11406-1/2008	H7N7	11.2	1,610,978	1,115,162	99.74
A/Ck/USA/PA/35154/1991	H1N1	12.4	1,807,144	1,316,695	99.62
A/Ck/USA/TX/G021090002/2002	H5N3	16.7	1,543,666	891,319	99.86
A/Ck/USA/CA/K0301417/2003	H6N2	11.6	1,022,630	895,629	99.92
A/Tk/USA/CO/169118-13/2002	H8N4	12.5	1,364,408	1,144,949	99.94

^1^Cycle threshold.

### 3.2 Alternative RT-PCR kits provided higher minimum read depth in polymerase segments

In our efforts to optimize the RT-PCR conditions for more efficient amplification of the polymerase genes, we focused on refining the amplification protocol by comparing different RT-PCR kits. To achieve this, we selected two alternative one-step RT-PCR kits: SSIV with the Platinum SuperFi DNA Polymerase and LS with the Q5 Hot Start High-Fidelity, which are more inhibitor-resistant and have a lower error rate compared to the SSIII kit with the Platinum *Taq* Polymerase recommended in Nanopore’s Ligation Sequencing Influenza Whole Genome Protocol. We further refined the RT-PCR thermocycling conditions for the alternative RT-PCR kits, as they require higher hot-start activation, higher annealing temperature, and shorter annealing time according to the manufacturer’s recommendations. Different RT times, annealing temperature and times were tested (data not shown). The optimal RT-PCR conditions for both alternative kits are provided at dx.doi.org/10.17504/protocols.io.bp2l62r15gqe/v1 (Goraichuk et al., 2024b).

**Figure 1:**
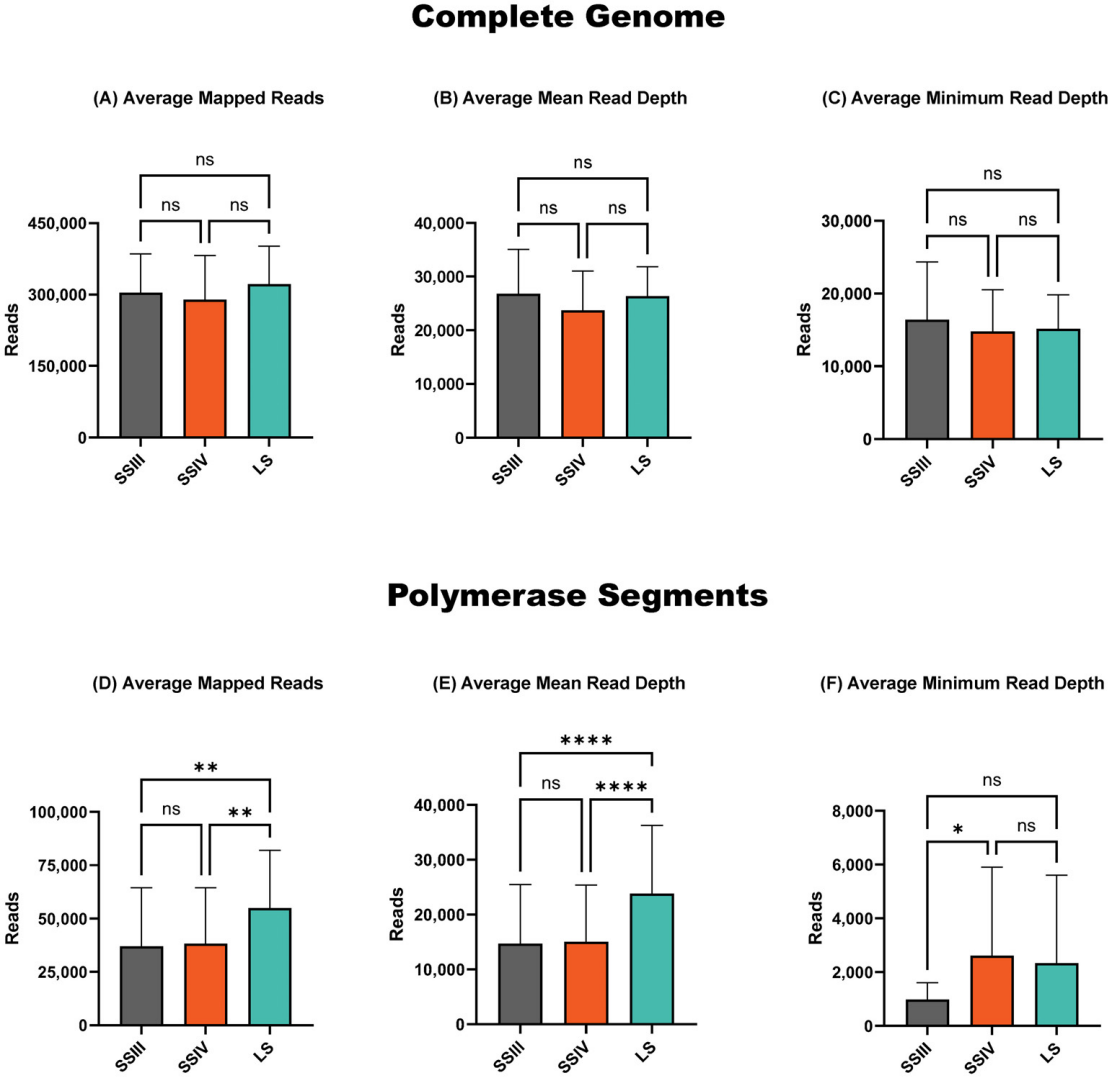
Sequencing summary for comparison of RT-PCR kits' performance on complete genome and polymerase segments. Average mapped avian influenza A reads for the complete genome **(A)** and polymerase segments **(D)** of eight different influenza viruses. Average avian influenza A genome mean read depth for the complete genome **(B)** and polymerase segments **(E)**. Average avian influenza A minimum read depth for the complete genome **(C)** and polymerase segments **(F)**. *P*-value is defined as follows: *p ≤ 0.05, **p ≤ 0.01, ****p ≤ 0.0001.

There were no significant differences between the compared RT-PCR kits in the average total number of influenza reads (**Figure 1A**), mean read number (**Figure 1B**), and minimum depth (**Figure 1C**) of reads mapped across the complete influenza genome. However, when examining the polymerase segments (PB2, PB1, and PA) separately, which typically exhibit the lowest minimum read depth, the alternative LS RT-PCR kit provided a significantly higher total and mean number of influenza reads (**Figures 1D, E**). Conversely, the minimum read depth in the polymerase segments was significantly higher (*p* < 0.05) for amplicons generated with the SSIV RT-PCR kit (**Figure 1F**).

Overall, both alternative RT-PCR kits outperformed the ONT-recommended SSIII kit. The LS kit produced a higher average number of sequenced reads (*p* < 0.01) and a higher mean read depth (*p* < 0.0001). However, it exhibited slightly lower minimum read depth coverage in the polymerase segments compared to the SSIV kit, though this difference was not statistically significant. Based on these findings, we selected the SSIV kit for further evaluation, as it provided the highest minimum read depth in the polymerase segments (**Figure 1F**).

### 3.3 Alternative Opti primers provided higher minimum read depth in polymerase segments

Next, we evaluated the performance of two primer sets: the ONT-recommended Tuni set and the alternative Opti set, validating them in conjunction with the ONT-recommended SSIII RT-PCR kit and the alternative SSIV kit, which had previously demonstrated superior results in minimum read depth coverage for polymerase segments. After testing various conditions (data not shown), we determined that an annealing temperature of 66°C for Tuni primers and 67°C for Opti primers was optimal for the alternative SSIV kit (**Supplementary Table 1**).

**Figure 2:**
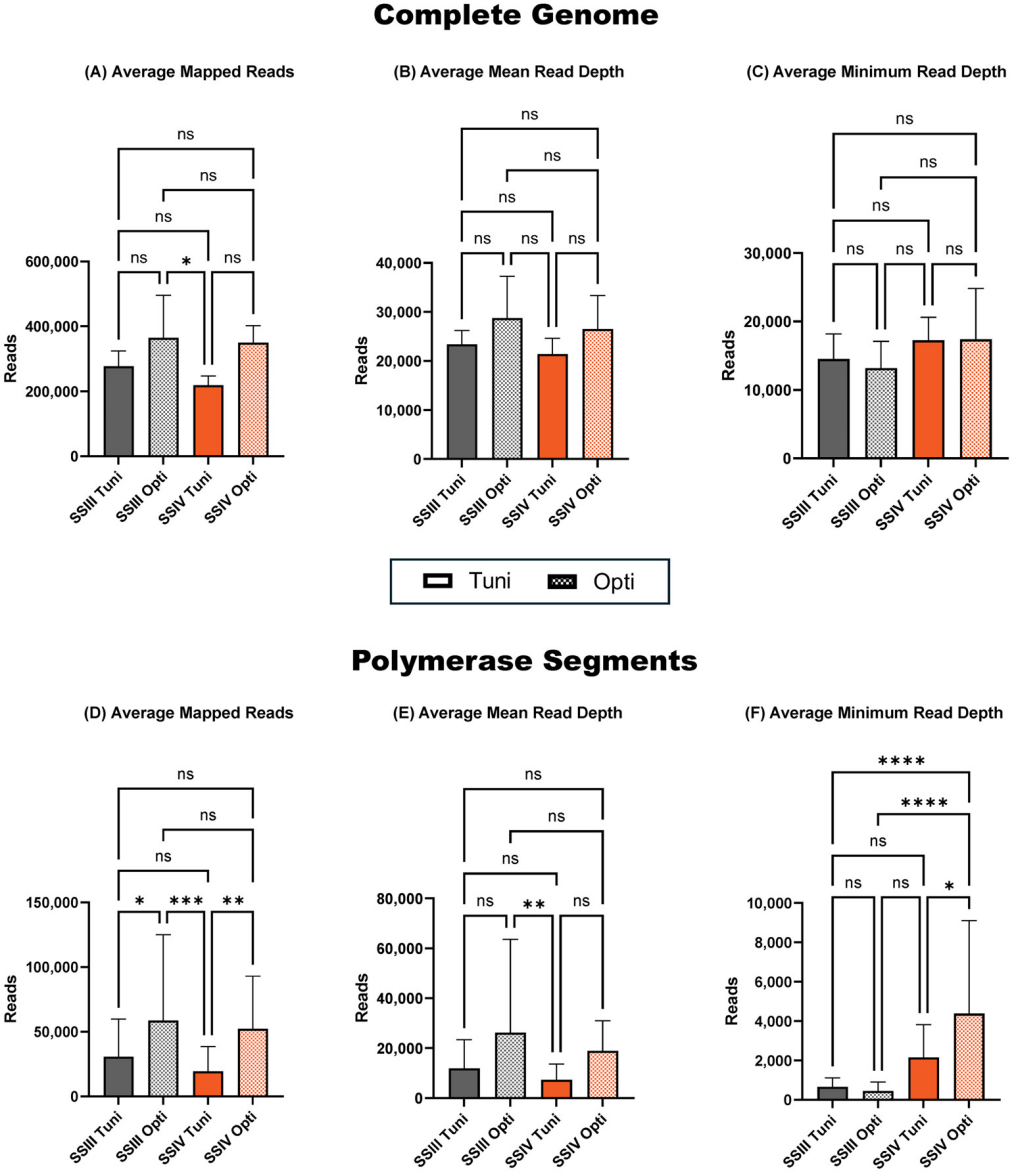
Sequencing summary for comparison of Tuni and Opti primer sets' performance on complete genome and polymerase segments. Average mapped avian influenza A reads for the complete genome **(A)** and polymerase segments **(D)** of six different avian influenza viruses. Average avian influenza A genome mean read depth for the complete genome **(B)** and polymerase segments **(E)**. Average avian influenza A minimum read depth for the complete genome **(C)** and polymerase segments **(F)**. *P*-value is defined as follows: *p ≤ 0.05, **p ≤ 0.01, ***p ≤ 0.001, ****p ≤ 0.0001.

Although non-significant, the Opti primer set yielded the highest average total and mean number of mapped reads across the complete genome, regardless of the RT-PCR kit used (**Figures 2A, B**). The average minimum read depth across all segments was higher in samples prepared with an alternative SSIV RT-PCR kit, regardless of the primer set used (**Figure 2C**). In the polymerase segments (PB2, PB1, and PA), there was no significant difference in the total and mean read number of reads between RT-PCR kits (**Figure 2D**). However, when comparing primer sets, the alternative Opti set was superior, providing a statistically significant increase in conjunction with both SSIII and SSIV (*p* < 0.05 and *p* < 0.01, respectively) in the average total number of sequenced reads. A similar impact was observed in the average mean read depth across polymerase segments (**Figure 2E**) with the alternative Opti primer set compared to the ONT-recommended Tuni primer set in both SSIII and SSIV RT-PCR kits, although not statically significant. The average minimum read depth of polymerase segments was significantly higher in samples prepared with the alternative Opti primer set in conjunction with the alternative SSIV RT-PCR kit compared to all other combinations (**Figure 2F**). This combination was selected for further evaluation of purification kits.

Notably, the SSIV RT-PCR kit not only increased the minimum read depth of the polymerase segments but also substantially improved the minimum read depth of the HA and NA segments, which are critical for influenza subtyping (**Supplementary Figure 1**).

### 3.4 Alternative purification Kit 4 was superior in filtering short reads

Next, we focused on evaluating different purification methods to remove short reads that could originate from defective interfering particles, potentially causing known U-shaped read coverage of polymerase segments. For this, we compared two magnet bead-based kits (Kit 1 and Kit 2) and two column-based kits (Kit 3 and Kit 4). The elution volume for different purification kits varied due to the manufacturer’s recommendations. However, after normalizing the obtained quantities, we found that Kit 1 provided the highest quantity of purified amplicons, followed by Kit 3 and Kit 2, while Kit 4 yielded the lowest quantity – averaging almost 5.7 times lower amount compared to unpurified samples (**Table 4**). Regarding quality, all purification kits performed well demonstrating A260/A280 absorbance ratio values of 1.8-2.0, which indicates a pure DNA sample. However, Kit 2 and Kit 4 were superior, with an average A260/A280 of 1.8, indicating optimal purity.

**Table 4:** Summary of purification quality before NGS library preparation.

Sample	Quantity - Qubit 1x HS, ng/µl ^1^					Quality - Nanodrop, A260/A280					Average Length - Tape Station, bp				
	Unpurified	Kit 1	Kit 2	Kit 3	Kit 4	Unpurified	Kit 1	Kit 2	Kit 3	Kit 4	Unpurified	Kit 1	Kit 2	Kit 3	Kit 4
H5N1	112	100.8	47.8	96	23.5	1.79	1.86	1.80	1.86	1.81	1356	1409	1397	1413	1796
H7N3	120	127.2	40.6	116	17.9	1.79	1.88	1.80	1.88	1.83	1330	1383	1413	1406	1725
H7N7	82.4	70.2	36.4	64.4	12.8	1.81	1.85	1.80	1.85	1.79	1006	1087	1116	1087	1533
H1N1	106	85.8	39.6	81.2	18.6	1.80	1.85	1.79	1.86	1.80	1278	1378	1377	1298	1704
H6N2	106	88.2	41.1	91.4	19.0	1.79	1.85	1.80	1.85	1.79	1209	1349	1320	1256	1712
H8N4	112	91.8	39.3	108	20.6	1.80	1.85	1.80	1.86	1.81	1468	1551	1496	1503	1929
Average	313.3	94	40.8	92.8	18.8	1.80	1.86	1.80	1.86	1.80	1274.5	1359.5	1353.2	1327.2	1733.2

^1^Concentrations after normalization to equal volume.

**Figure 3:**
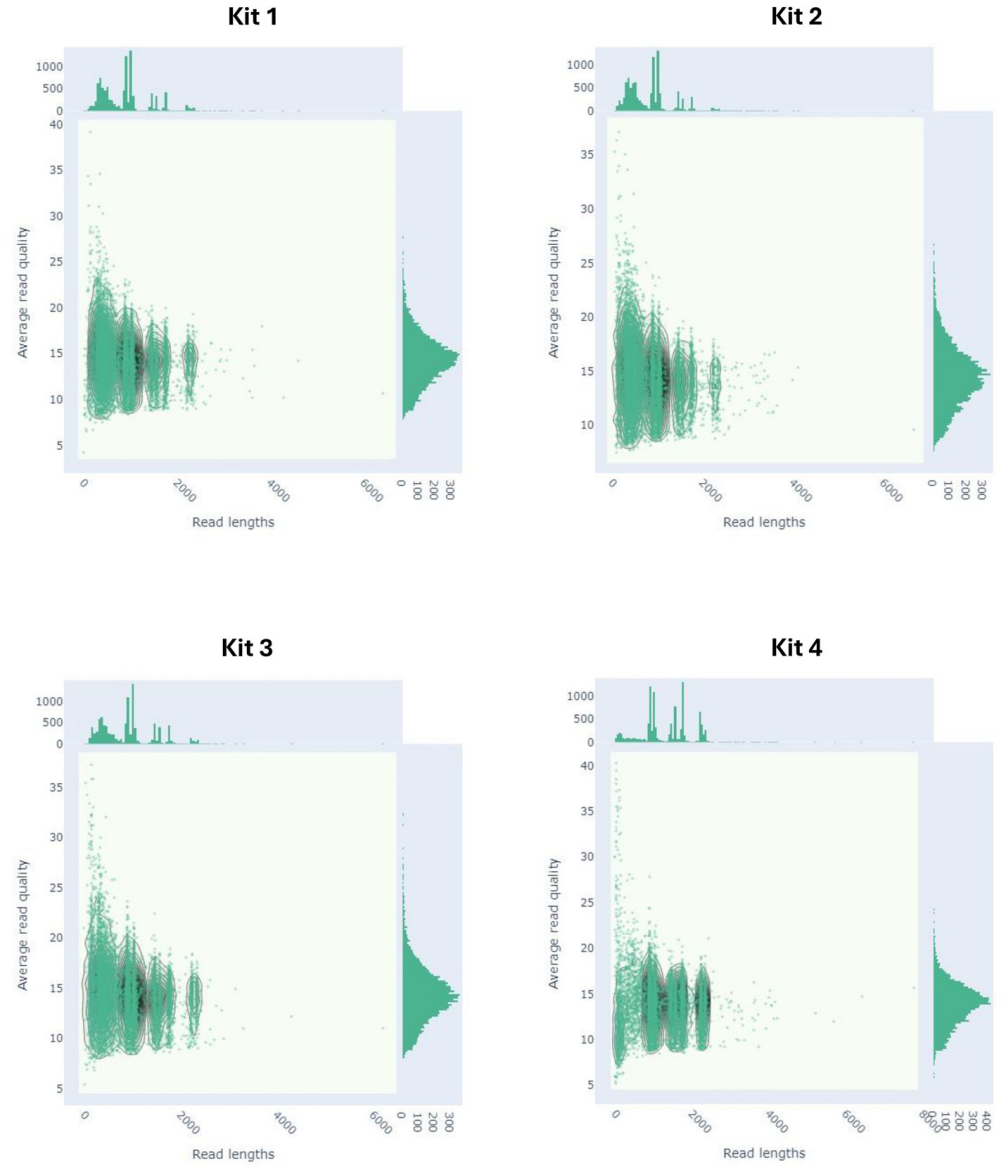
Read length vs average read quality kernel density estimation distribution plot of sequenced reads prepared with four different purification kits.

The average length distribution measured on the Tape Station indicated that all purification kits effectively eliminated smaller fragments, subsequently increasing the average length of the purified amplicons. Notably, Kit 4 demonstrated a substantial increase in average length compared to the other kits (**Table 4**). This improvement was also evident in the electropherogram of amplicons distribution before library preparation (**Supplementary Figure 2**) and further confirmed by the length distribution of sequenced reads (**Figure 3**).

Purification with Kits 1, 2, and 3 demonstrated that the majority of reads were still below 700 bp, which are considered non-targeted, as the shortest influenza segment is nearly 900 bp. In contrast, purification with Kit 4 effectively removed most of these untargeted reads, resulting in a shift in the read distribution towards longer influenza reads. Despite Kit 4 yielding the least number of reads, these reads had significantly higher median and mean read lengths, as well as a higher N50 value, indicating a successful reduction of shorter reads (**Table 5**).

**Table 5:** Summary statistics of ONT sequencing run of libraries prepared with four purification kits.

Purification Kit	Raw Reads	Median Read Length	Mean Read Length	Mean Read stdev	N50 ^1^	Mean Quality	Median Quality
Kit 1	2,031,438	904.0	900.4	523.0	1,043.0	13.1	14.4
Kit 2	1,722,942	893.0	846.3	507.4	1,040.0	13.1	14.4
Kit 3	1,648,596	906.0	895.2	538.5	1,046.0	13.1	14.3
Kit 4	1,042,050	1,268.0	1,327.1	675.9	1,747.0	12.7	14.0

^1^N50 represents the N50 length of all ONT reads followed by the number of reads constituting 50% of the length of all ONT reads.

**Figure 4:**
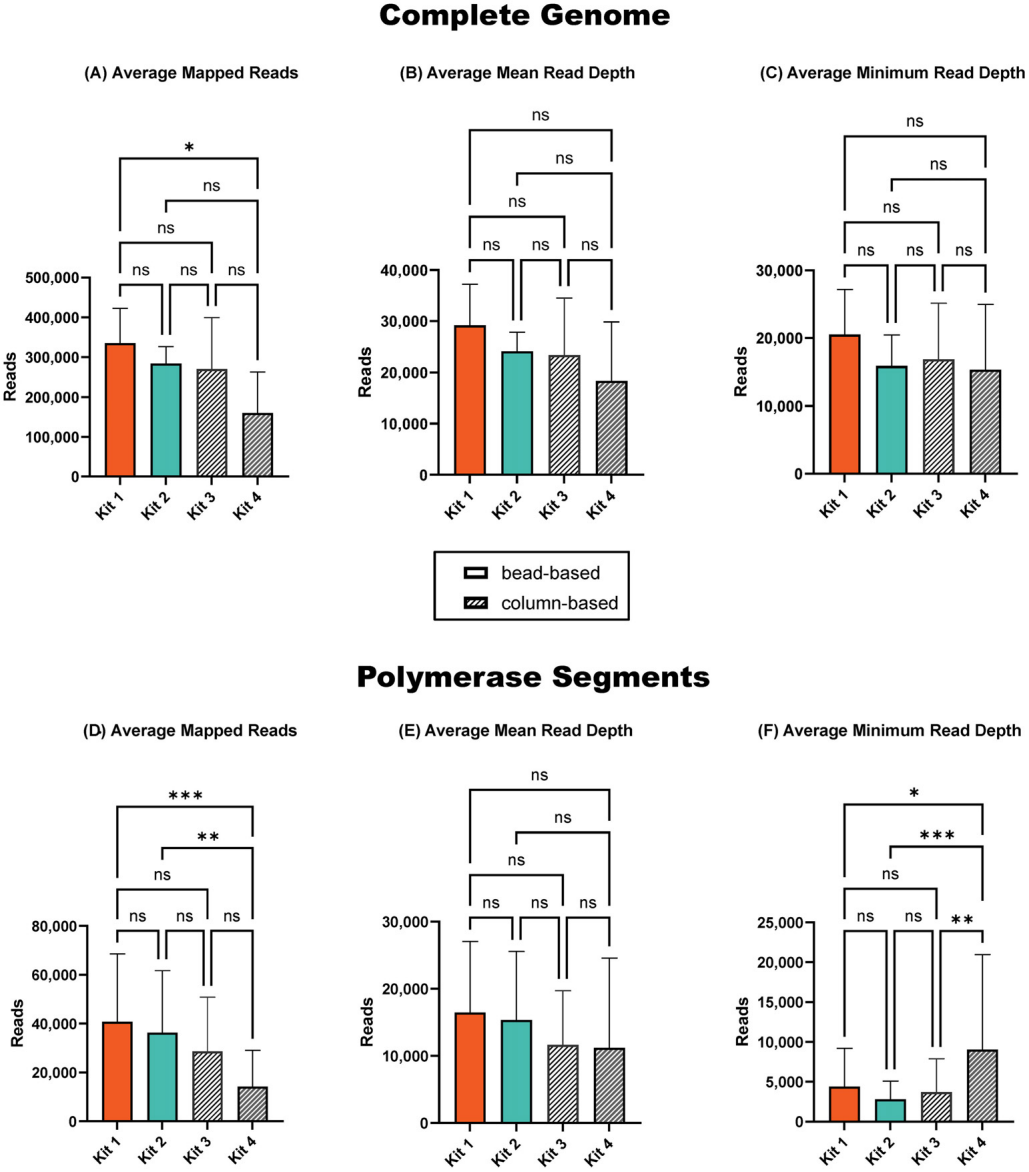
Sequencing summary for comparison of four amplicon purification kits' performance on complete genome and polymerase segments. Average mapped avian influenza A reads in the complete genome **(A)** and in polymerase segments **(D)** of six influenza viruses. Average avian influenza A genome mean read depth in complete genome **(B)** and in polymerase segments **(E)**. Average avian influenza A minimum read depth in complete genome **(C)** and in polymerase segments **(F)**. *P*-value is defined as follows: *p ≤ 0.05, **p ≤ 0.01, ***p ≤ 0.001.

The average number of sequenced reads was significantly lower (*p* < 0.05) after purification with Kit 4 compared to the ONT-recommended and commonly used Kit 1 (**Figure 4A**). However, the average total number of mapped reads, as well as the average mean and minimum read depth across the complete genome, did not show significant fluctuations between purification kits (**Figures 4A–C**). The polymerase segments, however, were more significantly impacted by the purification kits. Specifically, Kit 4 resulted in a significantly lower number of average sequenced reads (**Figure 4D**), while providing significantly higher minimum read depth coverage compared to all other purification kits (**Figure 4F**). The average mean read depth did not show notable variations between purification kits (**Figure 1E**).

The decrease in the total number of sequenced reads with Kit 4 was due to the removal of abundant short reads. This reduction in short reads allowed for a higher proportion of long reads to be sequenced, thereby increasing the minimum read depth of polymerase segments. The shift in read distribution is clear in the influenza genome read coverage plot (**Supplementary Figure 3**), demonstrating a more uniform coverage across the polymerase segments after purification with Kit 4, contrasting with the results from the other purification kits where the U-shaped distribution persisted. Additionally, Kit 4 offers significant time savings with 2-minute centrifugation and 1-minute incubation, compared to the recommended ONT kit’s 22-minute incubation. Automation with alternative Kit 2 was comparable to the manual purification.

**Figure 5:**
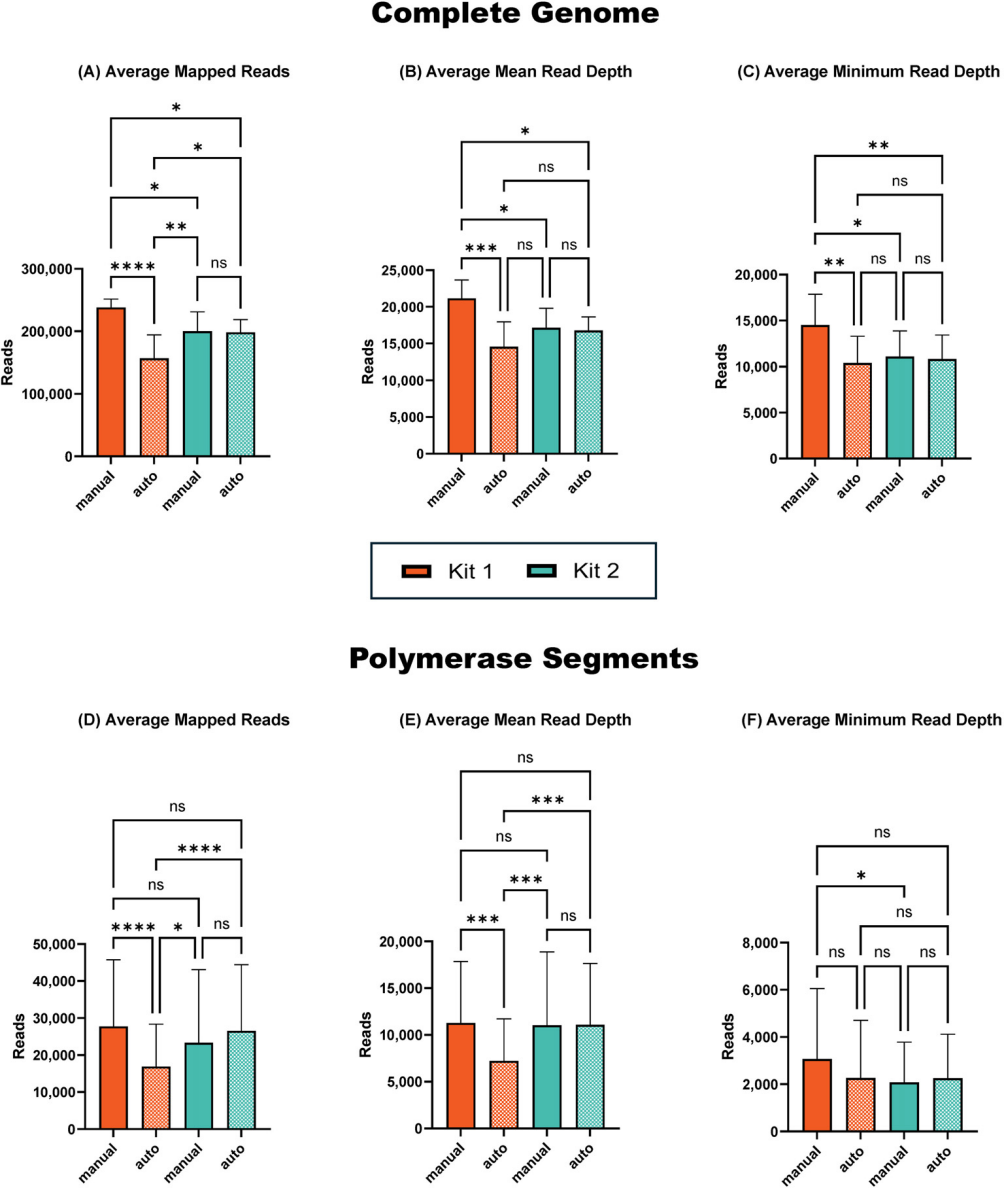
Sequencing summary for comparison of manual and automated amplicon purification performance on complete genome and polymerase segments. Average mapped avian influenza A reads in the complete genome **(A)** and in polymerase segments **(D)**. Average avian influenza A genome mean read depth in complete genome **(B)** and in polymerase segments **(E)**. Average avian influenza A minimum read depth in complete genome **(C)** and in polymerase segments **(F)**. *P*-value is defined as follows: *p ≤ 0.05, **p ≤ 0.01, ***p ≤ 0.001, ****p ≤ 0.0001.

To improve time efficiency for processing a high number of samples, we compared the performance of two bead-based kits (Kit 1 and Kit 2) in manual and automated purification. Our findings demonstrated that the total number of viral reads sequenced across the complete genome decreased with automated purification compared to manual purification (**Figure 5A**). This decrease was statistically significant (*p* < 0.001) for purification using Kit 1, but not significant for Kit 2. Similarly, the decrease in mean read depth coverage (**Figure 5B**) and minimum read depth coverage (**Figure 5C**) across the genome were significant (*p* < 0.001 and *p* < 0.01, respectively) for Kit 1 but not significant for Kit 2. Overall, manual purification with Kit 1 provided superior results.

Interestingly, for the polymerase segments, we observed a similar decrease in total, mean, and minimum read coverage with automation using Kit 1 (**Figures 5D–F**), while there was an increase in reads after automation with Kit 2, although this increase was not statistically significant. Thus, while automation severely affected the performance of the ONT-recommended Kit 1, automation with Kit 2 not only provided comparable results to its manual use but also matched the performance of the manual use of Kit 1. This indicates that Kit 2 is more suitable for automated purification when aiming to maintain high-quality sequencing results while processing a large number of samples efficiently.

## 4 Discussions

Our study aimed to optimize the ONT Ligation Sequencing Influenza A Whole Genome protocol (Oxford_Nanopore_Technologies, 2024) by comparing different RT-PCR kits, primer sets, and purification methods, and evaluating the feasibility of automation for high-throughput sample processing. The results demonstrate significant improvements in the total number of sequenced reads, minimum read depth coverage, and elimination of short, untargeted reads, which are crucial for reliable sequencing data and analysis.

The high mutation rate of Influenza A, coupled with recurrent detection of avian influenza in mammals, underscores the necessity of studying SNPs to unravel potential markers of mammalian adaptation. Reliable SNP analysis requires complete genome sequences with adequate read depth coverage. The conservative termini of influenza enable the simultaneous amplification of all eight segments, but achieving sufficient read depth coverage, especially for the longer polymerase segments (PB2, PB1, PA), remains challenging (Saira et al., 2013; Lee et al., 2016). These segments often exhibit U-shaped read coverage patterns with higher coverage at the 5’ and 3’ termini but lower coverage in the central regions (Van den Hoecke et al., 2015; Wang et al., 2015). This pattern is likely due to the presence of defective interfering particles, which are shorter sequences derived mainly from the polymerase segments that share the conserved termini but lack the central part of the sequence (Davis and Nayak, 1979; Davis et al., 1980; Ferreri et al., 2019; Świętoń et al., 2020). Although these shorter reads can be bioinformatically filtered post-sequencing to remove U-shaped coverage, this does not solve the problem of inadequate read depth coverage of longer segments. The initial sequencing of numerous short reads reduces the capacity to capture longer, full-length segments, resulting in insufficient read depth for these critical segments. Therefore, eliminating short reads before sequencing is essential to ensure adequate coverage and reliable downstream analysis. To address these challenges, we began with the ONT Ligation Sequencing Influenza A Whole Genome V14 protocol (Oxford_Nanopore_Technologies, 2024) as our baseline for optimization and verified our results using ONT sequencing. However, the optimized amplicon-targeted influenza A whole-genome sequencing protocol is also applicable to other short- and long-read NGS platforms. While amplicons after purifications are ready for library preparation for long-read platforms (e.g., ONT or Pacific Biosciences), for short-read platforms (e.g., Illumina, MGI, Singular Genomics, Ultima Genomics), an additional fragmentation step, which is typically part of the DNA library preparation protocol, would be necessary.

Previous studies have demonstrated the importance of selecting appropriate RT-PCR kits and primer sets to achieve uniform coverage across all segments (Wüthrich et al., 2019; Ip et al., 2023; Vereecke et al., 2023). Our study corroborated these findings, showing that the substitution of the ONT-recommended SSIII RT-PCR kit with alternative kits resulted in notable increases in total viral reads and minimum read depth coverage of polymerase segments. Particularly, the SSIV kit, containing the Platinum SuperFi DNA polymerase, provided the highest minimum read depth coverage, while the LS kit with the Q5 Hot Start High-Fidelity DNA Polymerase also yielded higher average read numbers and mean read depth coverage compared to the ONT-recommended SSIII kit. The LS kit is also a cost-effective alternative, with the cost per sample being 2.5 times lower compared to the SSIV kit and 2.2 times lower compared to the ONT-recommended SSIII, making it a practical option for large-scale sequencing projects where budget constraints are a consideration. Overall, both alternative RT-PCR kits demonstrated improved performance, underscoring their potential utility as viable alternatives to the SSIII kit. However, we tested only two commonly used RT-PCR kits, so there is room for incorporating other RT-PCR kits with different DNA polymerases into this protocol that can provide comparable or superior results. It is important to note that when evaluating new RT-PCR kits, adjusting thermocycling conditions is crucial, as the optimal conditions can vary significantly between different polymerases.

The substitution of the ONT-recommended Tuni primer sets with the alternative Opti primer set also demonstrated superior performance, yielding higher average read numbers and significantly improving the number of sequenced reads in polymerase segments, regardless of the RT-PCR kit used. This suggests that the Opti primers, while similar in length and containing conserved influenza termini regions, outperform the Tuni primers probably due to differences in their tail sequences. It is possible that the Tuni tails might anneal to non-influenza RNA/DNA, providing less targeted influenza amplification.

Purification of amplicons prior to sequencing is a critical step in enhancing read depth coverage and eliminating short, untargeted reads. The use of magnetic bead-based purification methods, such as AMPure XP, has been shown to improve sequencing quality by removing small fragments that can interfere with the sequencing process (Quail et al., 2009). To evaluate if alternative purification kits could offer additional benefits in terms of efficiency and cost-effectiveness, we tested three different purification kits. Our results demonstrated that, among all tested purification kits, Kit 4 was particularly effective in removing these short reads which was further reflected in the lowest concentrations observed after purification. The cost of Kit 4 is $2.86 per sample, which is comparable to the ONT-recommended Kit 1, priced between $0.80 and $3.90 per sample based on the volume of reagent purchased. Therefore, Kit 4 not only enhances the quality of sequencing data but also improves the efficiency and cost-effectiveness of the purification process, making it a valuable option for high-throughput sequencing applications. Moreover, Kit 4 offers significant time savings with 2-minute centrifugation and 1-minute incubation, compared to the ONT-recommended and commonly used Kit 1’s 22-minute incubation. Notably, the impact of purification kits on read depth coverage was more pronounced in the polymerase segments than in the complete genome, confirming our hypothesis that the abundant presence of short reads in sequencing libraries can reduce the minimum read depth of longer segments. While yielding fewer total reads, Kit 4 provided significantly higher minimum read depth coverage for the polymerase segments compared to all other tested kits, resulting in a substantial increase in the average read length and more uniform coverage across the genome by subsequently eliminating the U-shaped read distribution in the polymerase segments. Despite yielding the lowest number of sequencing reads Kit 4’s reads had significantly higher median and mean lengths, as well as a higher N50 value, indicating a successful reduction of shorter reads. This improvement was evident in the electropherogram of amplicons before sequencing and later confirmed by the length distribution of sequenced reads. Therefore, visualization of purified amplicons can be used as an additional QC verification step before expensive library preparation and sequencing in cases when new purification methods are tested for the elimination of short, untargeted reads. It is important to be cautious in interpreting results, as Kit 4 demonstrates that a significantly lower total number of sequenced reads of polymerase segments does not imply inferior results; rather, when considering the minimum read depth, Kit 4 provided significantly higher coverage.

Amplicon purification automation was explored to enhance efficiency in processing large numbers of samples. While automation with Kit 1 significantly reduced the number of reads and mean read depth coverage across all segments, automation with Kit 2 provided comparable results to its manual use. As for the minimum read depth coverage of polymerase segments, manual purification with Kit 1 provided the overall best results across the genome. However, they were comparable to automation with Kit 2. This indicates that Kit 2 is a viable option for automated high-throughput processing without compromising sequencing quality. The ability to maintain high-quality sequencing results with automation is crucial for scaling up surveillance and research efforts, particularly in response to emerging influenza threats.

Overall, our optimized protocol, which incorporates alternative substitutions for the RT-PCR kit, primer set, and amplicon purification kit, provides superior read depth coverage and effectively eliminates short, untargeted reads, subsequently increasing the minimum read depth coverage of polymerase segments. Additional purification automation assessments offer a feasible solution for high-throughput sample processing, maintaining the quality of manual purification methods. These advancements contribute to more reliable and efficient influenza A whole genome sequencing, crucial for studying markers of mammalian adaptation and improving surveillance of avian influenza viruses. It is important to note that consensus sequences generated on ONT and Illumina platforms didn’t have any nucleotide differences. Furthermore, this optimized Nanopore protocol is applicable across different NGS platforms, offering flexibility in selecting the platform that best aligns with the specific experimental needs for comprehensive influenza whole-genome studies. The refined SSIV and LS RT-PCR protocols can be found at dx.doi.org/10.17504/protocols.io.bp2l62r15gqe/v1 (Goraichuk et al., 2024b).

The findings from this study have implications for influenza virus research and public health surveillance. By enhancing the accuracy and efficiency of whole genome sequencing, our optimized protocol facilitates better detection and characterization of influenza viruses, including potential zoonotic strains. This is particularly important in the context of increasing detections of avian influenza viruses in mammals, where understanding markers of mammalian adaptation is critical for predicting and preventing potential pandemics.

In conclusion, the combination of advanced RT-PCR kits, optimized primer sets, effective purification methods, and feasible automation provides a robust framework for influenza A virus sequencing. Both the SSIV and LS RT-PCR kits, alongside the Opti primers and Kit 4 purification method, can be considered preferred alternatives to the current ONT protocol, offering improved read depth coverage, sequencing quality, and a much shorter and cheaper protocol, saving time and cost, and increasing overall efficiency. The optimized method is detailed in the provided protocol (Goraichuk et al., 2024b). Future studies should continue to refine these methods and explore their application to other viral pathogens, further enhancing our ability to monitor and respond to infectious disease threats.

## Data Availability

The data presented in the study are deposited in the GenBank database under accession numbers PQ060363-PQ060365, PQ064115-PQ064122, PQ064136-PQ064143, PQ064247-PQ064254, PQ064267-PQ064274, PQ064551-PQ064558, PQ106540-PQ106540, EU735794, EU735796, EU735799-EU735801, MH342039. Illumina and ONT alignment BAM files were deposited into the NCBI Sequence Read Archive under BioProject PRJNA1173216 (https://www.ncbi.nlm.nih.gov/bioproject/PRJNA1173216).
