# Supplementary material for: Improved influenza A whole-genome sequencing protocol
Source: Front Cell Infect Microbiol. 2024 Nov 28;14:1497278. doi: 10.3389/fcimb.2024.1497278 (PMC11635996; doi:10.3389/fcimb.2024.1497278)
Supplement: Supplementary file 1 [file Image1.pdf]

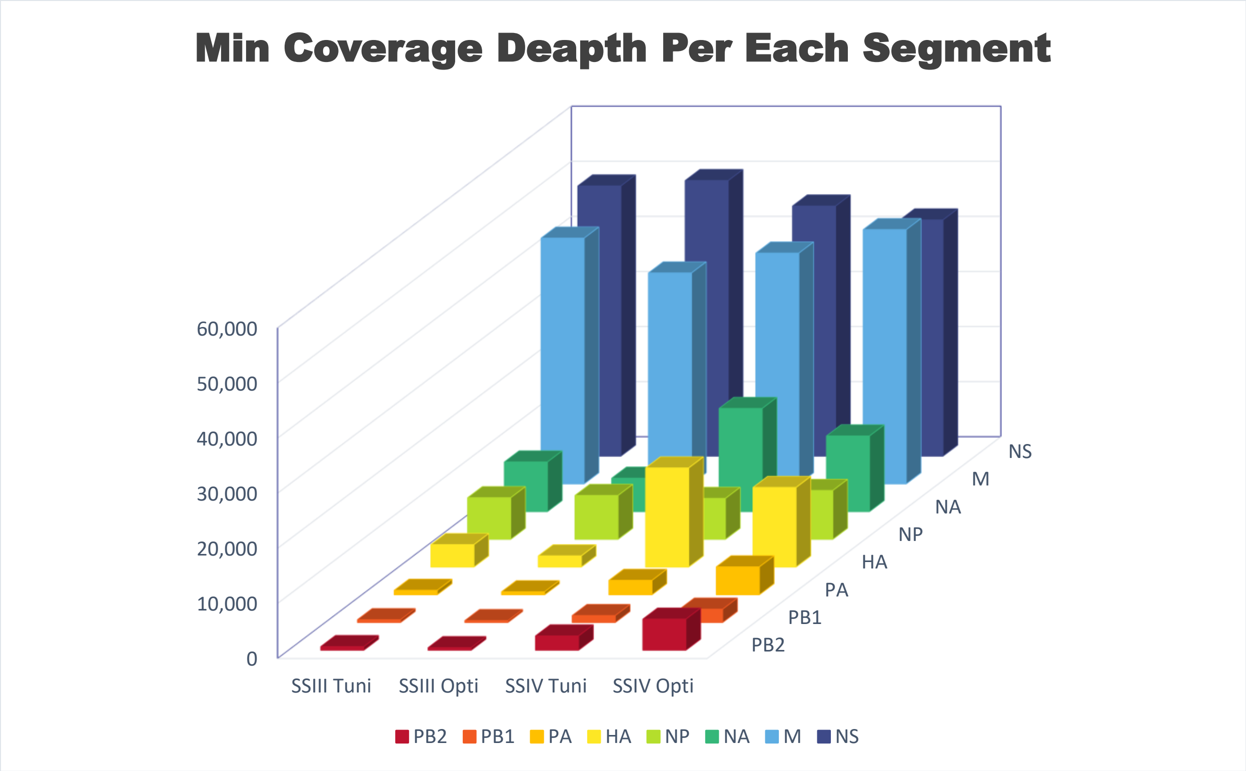

Supplementary Figure 1. Minimum read depth coverage per each segment for NGS libraries prepared using ONT-recommended SSIII and alternative SSIV RT-PCR kits with both Tuni and Opti primer sets.
