# Supplementary material for: Improved influenza A whole-genome sequencing protocol
Source: Front Cell Infect Microbiol. 2024 Nov 28;14:1497278. doi: 10.3389/fcimb.2024.1497278 (PMC11635996; doi:10.3389/fcimb.2024.1497278)
Supplement: Supplementary file 2 [file Image2.pdf]

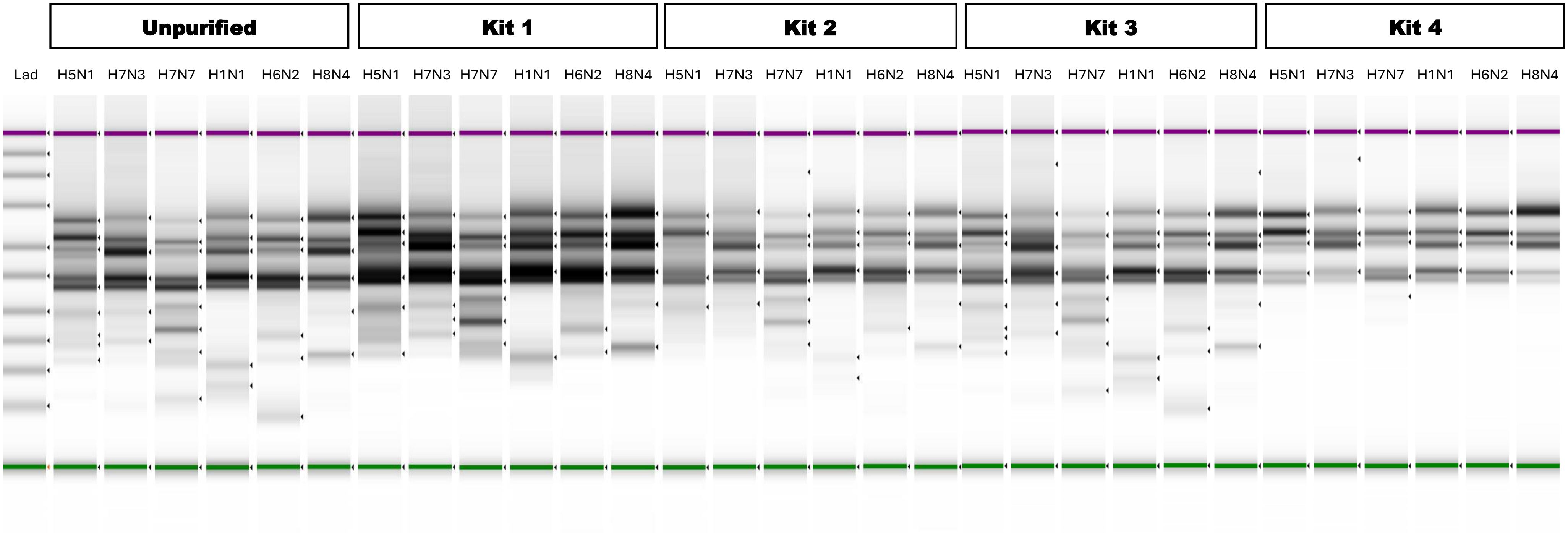

Supplementary Figure 2. TapeStation electropherogram of amplicons read length distribution purified with four different kits.
