# Supplementary material for: Improved influenza A whole-genome sequencing protocol
Source: Front Cell Infect Microbiol. 2024 Nov 28;14:1497278. doi: 10.3389/fcimb.2024.1497278 (PMC11635996; doi:10.3389/fcimb.2024.1497278)
Supplement: Supplementary file 3 [file Image3.pdf]

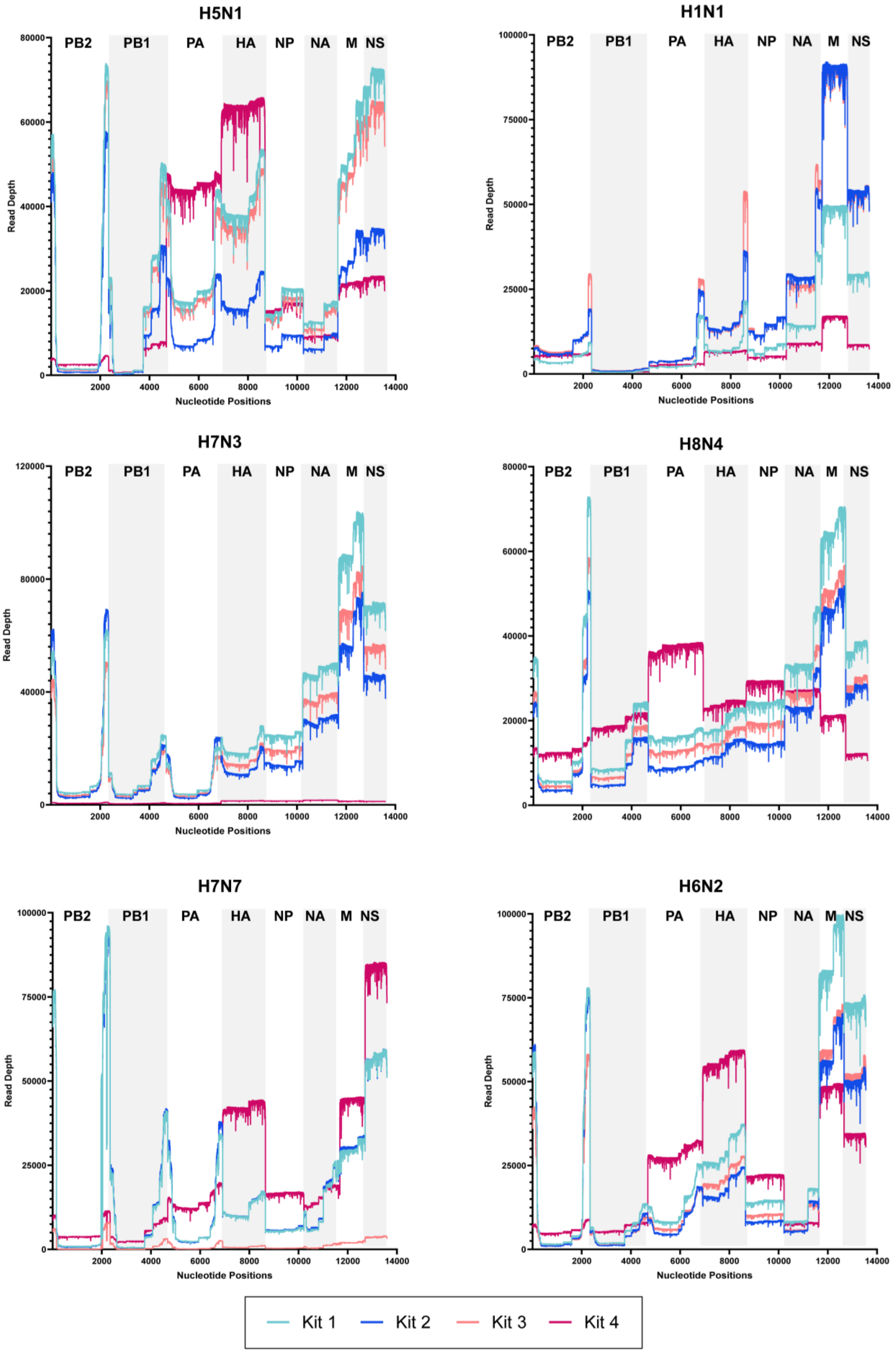

Supplementary Figure 3. Segment read depth coverage of influenza genomes (H5N1, H7N3, H7N7, H1N1, H5N3, and H6N2) purified with different kits. Sequencing reads obtained on the ONT platform were mapped to the reference genome *de novo* assembled from reads obtained on the Illumina platform.
