# Supplementary material for: Improved influenza A whole-genome sequencing protocol
Source: Front Cell Infect Microbiol. 2024 Nov 28;14:1497278. doi: 10.3389/fcimb.2024.1497278 (PMC11635996; doi:10.3389/fcimb.2024.1497278)
Supplement: Supplementary file 4 [file Table1.pdf]

Supplementary Table 1. Summary of RT-PCR thermocycling conditions, master mix, and primer sets used.

Primer Mix

*To prepare the **TUNI primer mix**, add primer stock solution so that there is 4: 1:5 ratio of Tuni12:Tuni12.4:Tuni13 primers. Sample calculation below:

Name	Sequence
Tuni 12	ACG CGT GAT CAG CAA AAG CAG G
Tuni 12.4	ACG CGT GAT CAG CGA AAG CAG G
Tuni 13	ACG CGT GAT CAG TAG AAA CAA GG

Primer	Volume
nuclease-free water	90 µl
Tuni 12 (100 µM)	4 µl
Tuni 12.4 (100 µM)	1 µl
Tuni 13 (100 µM)	5 µl
**TOTAL**	**100 µl**

*To prepare the **OPTI primer mix**, add primer stock solution so that there is 0.35: 0.65:1 ratio of Opti F1:F2:R primers. Sample calculation below:

Name	Sequence
Opti F1	GTT ACG CGC CAG CAA AAG CAG G
Opti F2	GTT ACG CGC CAG CGA AAG CAG G
Opti R	GTT ACG CGC CAG TAG AAA CAA GG

Primer	Volume
nuclease-free water	80 µl
Opti F1 (100 µM)	3.5 µl
Opti F2 (100 µM)	6.5 µl
Opti R (100 µM)	10 µl
**TOTAL**	**100 µl**

Thermocycling conditions

SuperScript III Platinum with TUNI primers

42°C	60	min	
94°C	2	min	
94°C	30	sec	
45°C	30	sec	5 cycles
68°C	3	min	
94°C	30	sec	
57°C	30	sec	31 cycles
68°C	3	min	
4°C	hold		

SuperScript III Platinum with OPTI primers

55°C	2	min	
42°C	90	min	
94°C	2	min	
94°C	15	sec	
45°C	30	sec	5 cycles
68°C	3.5	min	
94°C	15	sec	
63°C	30	sec	30 cycles
68°C	3.5	min	
68°C	10	min	
4°C	hold		

SuperScript IV with TUNI primers

55°C	2	min	
42°C	90	min	
98°C	2	min	
98°C	10	sec	
45°C	30	sec	5 cycles
72°C	3.5	min	
98°C	10	sec	
66°C	30	sec	30 cycles
72°C	3.5	min	
72°C	10	min	
4°C	hold		

SuperScript IV with OPTI primers

55°C	2	min	
42°C	90	min	
98°C	2	min	
98°C	10	sec	
45°C	30	sec	5 cycles
72°C	3.5	min	
98°C	10	sec	
67°C	30	sec	30 cycles
72°C	3.5	min	
72°C	10	min	
4°C	hold		
