# Supplementary material for: Improved influenza A whole-genome sequencing protocol
Source: Front Cell Infect Microbiol. 2024 Nov 28;14:1497278. doi: 10.3389/fcimb.2024.1497278 (PMC11635996; doi:10.3389/fcimb.2024.1497278)
Supplement: Supplementary file 5 [file Table2.pdf]

Supplementary Table 2. Summary statistics of four ONT sequencing runs from this study.

Run Description	Library Amount, fmol	Available Pores		Sequenced Reads, M	Demultiplexed Reads, M	Influenza Reads, M
		before	after			
RT-PCR comparison	20	1,630	523	10.21	7.51	7.32
Primers comparison	60	1,652	1,081	9.42	8.66	7.26
Purification comparison	60	1,547	876	8.61	7.71	6.30
Automation assessment	80	1,623	907	7.69	4.80	4.76
